# Retrospective analysis of the long-term therapeutic effectiveness and safety profile of rituximab in the treatment of mucous membrane pemphigoid in a German university center between 2008 and 2019

**DOI:** 10.3389/fimmu.2023.1180150

**Published:** 2023-04-18

**Authors:** Florian Bamberger, Inke R. König, Damian Gola, Detlef Zillikens, Christian D. Sadik

**Affiliations:** ^1^ Department of Dermatology, Allergy, and Venereology, Lübeck, Germany; ^2^ Institute of Medical Biometry and Statistics (IMBS), Lübeck, Germany; ^3^ Center for Research on Inflammation of the Skin (CRIS), University of Lübeck, Lübeck, Germany

**Keywords:** mucous membrane pemphigoid, rituximab, pemphigoid disease, autoantibodies, side-effects, B cell depletion, retrospective study, mucous membrane pemphigoid disease index

## Abstract

**Background:**

The B-cell-depleting anti-CD20 antibody rituximab (RTX) is often used as an adjuvant drug for the treatment of refractory cases of mucous membrane pemphigoid (MMP).

**Objective:**

This study aims to determine the therapeutic effectiveness and the safety profile of RTX in MMP.

**Methods:**

The medical records of all cases of MMP treated with RTX between 2008 and 2019 in our university medical center located in northern Germany, which specialized in autoimmune blistering skin diseases, were retrieved and systemically analyzed for treatment responses and potential adverse events over a median period of 27 months.

**Results:**

We identified 18 MMP patients who received at least one cycle of RTX to treat MMP. RTX was always used as an adjuvant treatment, and its application did not change concomitant treatments. Under treatment with RTX, 67% of the patients achieved an improvement in their disease activity within 6 months. This was also reflected in a statistically significant reduction in the *Mucous Membrane Pemphigoid Disease Index* (MMPDAI) activity score. The frequency of infections under RTX treatment increased only slightly.

**Conclusions:**

The use of RTX is associated with an attenuation of MMP in a large proportion of MMP patients in our study. At the same time, its application was not found to further increase the susceptibility of the most strongly immunocompromised population of MMP patients to opportunistic infections. Collectively, our results suggest that the potential benefits of RTX outweigh its risks in patients with refractory MMP.

## Introduction

Mucous membrane pemphigoid (MMP) is an antibody-mediated autoimmune disease. The most common autoantigens are the C-terminus of type XVII collagen (BP180), BP230, laminin 332, integrin α6β4, and type VII collagen (1). MMP differs from other pemphigoid diseases in that it primarily affects the mucosa of the conjunctiva, oral cavity, esophagus, nose, pharynx, larynx, trachea, anal canal, and genitalia (2). Scarring is another symptom of MMP unique to pemphigoid diseases. It mostly manifests at the eye and as strictures in the esophagus, pharynx, and larynx. Preventing the development of scarring is an important goal in the treatment of MMP and often requires a swiftly initiated, marked, and long-term maintained immunosuppression. Drugs used in the treatment of MMP, mostly in combination, include systemic and topical corticosteroids, mycophenolate mofetil, azathioprine, dapsone, intravenous immunoglobulins, cyclophosphamide, and rituximab (RTX) (2).

RTX is an anti-CD20 antibody designed to deplete B cells (3). In MMP, it is used off-label following the pharmacological rationale that depleting B cells to blunt the production of pathogenic autoantibodies should have beneficial effects. However, with clinical trials of adequate size on the impact of RTX on MMP still lacking, the effectiveness and safety of RTX in MMP have remained controversial. We have therefore retrospectively analyzed the long-term impact of rituximab on disease and adverse events in 18 MMP patients treated in our department specializing in pemphigoid diseases between 2008 and 2019.

## Materials and methods

The study was approved by the ethics committee of the University of Lübeck (20-130A). The medical records of all patients treated for MMP at the University of Lübeck between 2008 and 2019 were retrieved. Patients who received RTX at least once were identified, and their medical history before and after the first administration of RTX was examined by analyzing all medical records of in- and outpatient visits available.

Disease activity was categorized as “active disease,” “disease control,” “partial remission,” and “complete remission” according to the definitions of an international consensus conference (4). We modified this categorization to additionally distinguish “complete remission on therapy” and “partial remission on therapy” (**Supplementary Table S1**).

All side effects were recorded to detect potential adverse events associated with the administration of RTX. The period of 6 months after RTX administration was given special attention throughout all analyses of efficacy in MMP because the effect of RTX is supposed to reach its maximum at this time.

*Mucous Membrane Pemphigoid Disease Area Index* (MMPDAI) scores before and approximately 6 months after RTX were statistically analyzed using the Wilcoxon signed-rank test. Statistical significance was defined by a *p*-value of < 0.05. R version 4.1.1 was used for statistical analysis.

## Results

A total of 77 patients were treated for MMP as inpatients in our department between April 2008 and December 2018. Approximately 21 patients (27%) received at least one cycle of RTX, and 18 were followed up after the administration of RTX for at least 5 months between April 2008 and September 2019 (**Supplementary Table S2**). Those 18 patients were included in our detailed analysis. Ten of them received one, while eight had two cycles of RTX using the “rheumatoid arthritis protocol,” i.e., two administrations of 1 g of RTX within 2–4 weeks (5). Their demographics, pattern of organ involvement, autoantigens, and the treatments received prior to RTX are summarized in **Table 1** and **Supplementary Tables S3, S4**.

**Table 1:** Demographics, organ involvement, and therapies prior to RTX.

Gender (number of patients)	
Men	5 (27.8%)
Women	13 (72.2%)
Age (median)	
Age at diagnosis (years)	61.5 (range, 23–88)
Age at first RTX administration (years)	64.5 (range, 24–90)
MMP organ involvement (number and percentage of patients)	
Ocular involvement	6 (33.3%)
Oral involvement	15 (83.3%)
Nasal involvement	8 (44.4%)
Laryngeal involvement	1 (5.6%)
Genital involvement	4 (22.2%)
Anal involvement	2 (11.1%)
Cutaneous involvement	2 (11.1%)
**Disease duration (median) before first RTX administration (months)**	14.0 (range, 2–59)
Previous systemic therapies (number and percentage of patients)	
Corticosteroids	17 (94.4%)
Adjuvant therapy	16 (88.9%)
* Mycophenolic acid*	11 (61.1%)
* Azathioprine*	5 (27.8%)
*Intravenous immunoglobulin*	4 (22.2%)
*Protein A immunoadsorption*	1 (5.6%)
*Dapsone*	13 (72.2%)
*Cyclophosphamide*	2 (11.1%)
*Cyclosporine A*	1 (5.6%)

RTX, rituximab; MMP, mucous membrane pemphigoid.

The median time between the first diagnosis of MMP and the first cycle of RTX was 14 months (range, 2–59 months). In all 18 cases, RTX was applied as an adjuvant treatment together with corticosteroids. In total, 17 patients received at least one more immunosuppressive or immunomodulatory drug (**Table 1**; **Supplementary Table S3**).

**Figure 1:**
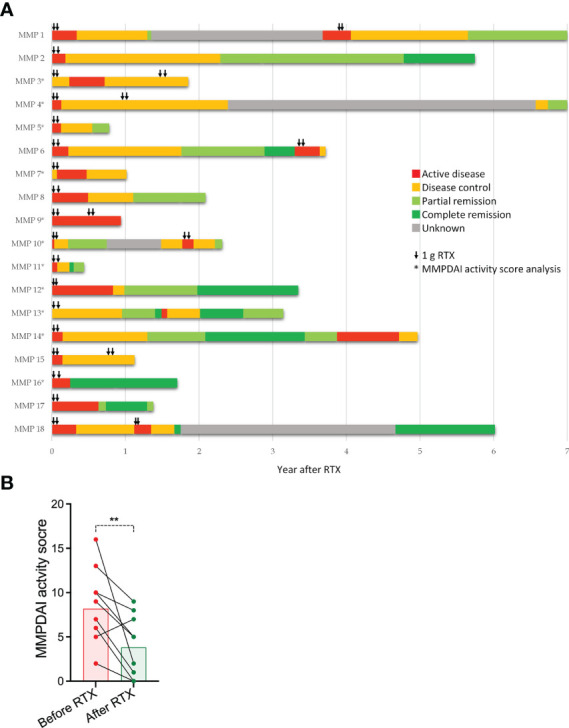
MMP disease activity before and in the years after the administration of RTX. **(A)** Individual course of MMP disease activity in 18 MMP patients starting with the first administration of RTX. Disease was categorized as “active disease,” “disease control,” “partial remission,” “complete remission,” and “unknown,” following the categorization system described in the Materials and methods section. Black arrows indicate the time points of the administration of 1 g of RTX. **(B)** Comparison of the MMPDAI activity score at the initiation of RTX and 6 months later. Each dot represents an individual patient (*n* = 11). The black lines connect the MMPDAI activity scores before and after the RTX for the individual patient. The MMPDAI before and after RTX was compared by a Wilcoxon signed-rank test (*p* = 0.0073).

The course of disease with the categorization of disease activity over a median period of 27 months after the first administration of RTX is compiled for each individual patient in **Figure 1A**. RTX was initiated in 15 patients with active disease and in three patients with controlled disease who showed no further improvement with first- and second-line therapy. In 12 patients, the administration of RTX was followed by a decrease in disease activity within 6 months. In two patients (MMP3/7), the disease aggravated during this time. During the time of follow-up of up to 7 years, 14 patients exhibited periods of partial or complete remission. In three patients (MMP10/11/16), a partial or complete remission was achieved within 6 months after RTX (**Figure 1A**). This was also reflected in the discontinuation of the dexamethasone pulses applied to all patients at the time of the initiation of RTX or in the extension of the time intervals between the individual pulses 6 months after the administration of RTX (**Supplementary Table S5**). There was no significant association between the response to RTX and autoantibody specificity or body sites affected by MMP (results not shown).

In 11 patients, the MMPDAI activity score (4) was assessed repeatedly before and after treatment with RTX, allowing a more objective comparison of disease activity at the initiation of RTX therapy (day 0) and approximately 180 days thereafter. This comparison revealed a statistically significant decrease from a median of 9 to a median of 5 (**Figure 1B**).

To assess the safety profile of RTX in our patient cohort, we systemically reviewed the patients’ records for medical conditions constituting potential RTX-induced adverse events. Our patient records allowed follow-up for up to 58 months before and 85 months after the first administration of RTX. This comparison showed that the frequency of potential adverse events did not change in our cohort after the administration of RTX (**Table 2**). Comparing the pattern and frequency of possible adverse events exclusively in the 12 months before and after the administration of RTX showed no differences except for a slight tendency towards more respiratory tract infections, with four patients developing newly respiratory tract infections in the first 12 months after RTX (**Supplementary Table S1**). Furthermore, only one patient (MMP8) developed a severe infection, specifically sepsis. None of the patients died in the year following the administration of RTX. No significant association was found between the number of infections during the 12 months after RTX and autoantibody specificity or sites affected by MMP (results not shown).

**Table 2:** Frequency of potential side effects.

	Therapy before RTX (*n* = 15)	Therapy after RTX (*n* = 18)	
Median documented time (months)^a^	5.9 (range, 1–58)	26.8 (range, 5–85)	
Documented adverse events	Number of patients	Number of patients	Median TAE (months)
**Infections**	9 (60%)^ibefore^	12 (67%)^iafter^	5.0 (range, 1–64)
**Eyes**	1 (7%)	1 (6%)^e^	13
Conjunctivitis	–	1 (6%)	44
Meibomitis	1 (7%)	–	
Ocular bacterial inflammation^b^	–	1 (6%)	13
**Respiratory system**	6 (40%)	8 (44%)^i^	4.5 (range, 1–64)
Upper respiratory infection	4 (27%)^c^	6 (33%)	5.5 (range, 3–64)
Tracheitis	–	1 (6%)^d^	39
Bronchitis	2 (13%)	1 (6%)	2
Pneumonia	0 (0%)	1 (6%)	1
**Gastrointestinal tract**	1 (6%)	2 (9%)	49.0 (range, 10–88)
Gastrointestinal infection	1 (6%)	2 (9%)^e^	
**Urinary tract**	4 (27%)	4 (22%)	10.5 (range, 1–26)
Urinary tract infection	4 (27%)	4 (22%)	
**Systemic**	0 (0%)	1 (6%)	6
Sepsis	–	1 (6%)^f^	
**Other**	2 (13%)^j^	–	–
Hepatitis B infection	1 (7%)	–	
Genital herpes	1 (7%)	–	
Pulpitis	1 (7%)	–	
**Liver**	0 (0%)	1 (6%)	36
Steatosis hepatis	–	1 (6%)	
**Musculoskeletal**	0 (0%)	4 (22%)^k^	1.5 (range, 0–10)
Osteoporosis	–	2 (11%)	25.5 (range, 10–41)
Osteopenia	–	1 (6%)	2
Myalgia	–	1 (6%)	0
Fibromyalgia syndrome	–	1 (6%)	1
Arthrosis	–	1 (6%)	11
**Neurological**	1 (7%)^l^	2 (11%)	33.5 (range, 5–62)
Apoplex	–	1 (6%)	62
Cerebral aneurysm	1 (7%)	–	
Polyneuropathy	1 (7%)	–	
Muscular cramps	–	1 (6%)^g^	5
**Mental disorders**	0 (0%)	1 (6%)	26
Depressive episodes	–	1 (6%)	
**Weight**	0 (0%)	1 (6%)	28
Cachexia	–	1 (6%)	
**Cardiovascular system**	1 (7%)	1 (6%)^m^	3
Reduced ejection fraction (30%)	–	1 (6%)	3
Orthostatic circulatory dysregulation	1 (7%)	–	
Left bundle branch block	–	1 (6%)	3
**Eyes**	0 (0%)	2 (11%)^n^	3.5 (range, 3–4)
Macular degeneration	–	1 (6%)	18
Macular foramen	–	1 (6%)	3
Vitreous opacity	–	1 (6%)	3
Progressive cataract formation	–	1 (6%)	4
**Hypersensitivity**	0 (0%)	1 (6%)	0
Asthma exacerbation	–	1 (6%)	
**Respiration**	0 (0%)	2 (11%)	6.5 (range, 0–13)
Dyspnoea	–	2 (11%)	

RTX, rituximab; TAE, time to adverse event (time until the first occurrence of the adverse event, based on the day of the first RTX administration of the first RTX cycle).

a Therapy before RTX: based on the first patient presentation at the Department of Dermatology, Allergology, and Venerology at the University Hospital Schleswig-Holstein (Lübeck) up to the first RTX administration. Therapy after RTX: based on the day of the first RTX administration of the first RTX cycle.

b Not more precisely defined.

c Includes ulcerating and granulating pharyngitis.

d With severe tracheal stenosis.

e Includes a patient with pangastritis and bulbitis.

f By port infection with Escherichia coli.

g Recurrent: (a) ^ibefore^, nine patients developed one or more infections: two patients developed bronchitis. Two patients developed a urinary tract infection. One patient developed an upper respiratory infection and a hepatitis B infection. One patient developed urinary tract infections and an upper respiratory infection. One patient developed meibomitis and a urinary tract infection. One patient developed genital herpes, an upper respiratory infection, gastrointestinal infection, and pulpitis. One patient developed an upper respiratory infection. (b) ^iafter^, 12 patients developed one or more infections: one patient developed an upper respiratory infection. One patient developed an upper respiratory infection. One patient developed an upper respiratory infection, a urinary tract infection, and a gastrointestinal infection. One patient developed pneumonia and an upper respiratory infection. One patient developed tracheitis with severe tracheal stenosis. One patient developed a gastrointestinal infection. One patient developed an upper respiratory infection and sepsis. Two patients developed a urinary tract infection. One patient developed bronchitis. One patient developed ocular bacterial inflammation and conjunctivitis. One patient developed urinary tract infections and an upper respiratory infection.

h One patient developed conjunctivitis some time after ocular bacterial inflammation.

i One patient developed an upper respiratory infection some time after pneumonia.

j One patient developed genital herpes and pulpitis in the documented time prior to RTX administration.

k One patient was diagnosed with arthrosis after osteoporosis. Another patient was found to have osteoporosis after myalgia.

l One patient developed a cerebral aneurysm and polyneuropathy in the documented time prior to RTX administration.

m In one patient, both a reduced ejection fraction and a left bundle branch block were found.

n Macular degeneration was found in one patient in addition to progressive cataract formation. Another patient was diagnosed with vitreous opacity in addition to a macular foramen. In some patients, several adverse events falling into one category have occurred. The adverse event with the shortest TAE among the respective patients was included in the calculation of the median TAE for the corresponding categories.

An analysis of 11 patients showed no difference in the peripheral blood leucocyte counts and the serum levels of the C-reactive protein (CRP) and liver enzymes 100 days before and after the first administration of RTX (results not shown).

## Discussion

We retrospectively analyzed the course of the disease in 18 MMP patients who received at least one cycle of RTX for MMP. The cohort of our 18 MMP patients appears representative of MMP, with its demographics and pattern of organ affection closely resembling those of other MMP patient cohorts previously published. After 6 months, when RTX is considered to have had its maximal effect, the severity of MMP was significantly reduced in 12 patients (67%), which was also indicated for a subgroup of our cohort by the MMPDAI activity score. However, as other therapeutics were continued after the administration of RTX, it is not possible to clearly distinguish to what extent this improvement was a result of the RTX-mediated depletion of B cells. However, with the treatment before and after RTX administration not changing in most patients and most patients having already been treated for a long time prior to RTX, it is likely that RTX contributed to this improvement. In six patients (33%), disease activity did not decrease or barely decreased, indicating that RTX might only be effective in a subgroup of patients. This suggests that the RTX-mediated depletion of B cells either does not reach the pathogenic autoreactive B-cell population in all patients or that the continuous production of autoantibodies is not required in a subgroup of MMP patients to sustain disease activity.

With 67% of our cohort responding to RTX, our response rate was similar to that recently reported in retrospective studies from academic dermatological centers in France and the Netherlands as well as in a systematic review, which retrieved the reports on 112 MMP patients (6–9). The latter found that the administration of RTX was associated with a complete resolution of disease in 70.5% of MMP patients within 8.7 months (6). In the two Dutch studies, 64% and 75% of patients with refractory MMP responded to rituximab with partial or complete remission (7, 8).

Bohelay et al. retrospectively analyzed the response of 109 patients with severe and/or refractory MMP to RTX in a French center (9). The outcomes were defined in this study, like in ours, following the definitions of Murrell et al. (4). The standard treatment regimen used in the French center differed from our protocols, e.g., in that it included more regular administrations of RTX. More specifically, patients received RTX every 6 months until complete remission was achieved or failure was declared. Patients who did not show an improvement 3 months after the first administration of RTX received another cycle of RTX at this point. When complete remission was achieved, the patients received another cycle of RTX before the drug was discontinued. All patients received dapsone or salazopyrine, which was the only adjuvant therapy in 106 of the 109 patients. Under this regimen, 85.3% of patients achieved complete remission after a median of two cycles of rituximab, i.e., within 1 year after the initiation of RTX, but the disease relapsed in 38.7% of these patients. Thus, while the response to the first cycle of RTX reported in this study was similar to our finding, the response rate over time was higher and the remission was more stable after several cycles of RTX than the outcomes in our study. Collectively, this suggests that several regular cycles of RTX are beneficial for MMP patients and lead to better outcomes. Importantly, like our study, Bohelay et al. did not find a substantial increase in the rate of adverse events under RTX either. The most common adverse events reported were diverse infections.

A strength of our study is that we can compare the change in disease activity of 11 MMP patients before and after RTX using the MMPDAI activity score. Although calculating this score is laborious, it should become part of the clinical routine in all medical centers to extend the capabilities for insightful clinical studies in this field.

Our study did not detect any change in adverse events potentially associated with RTX, except for a slight increase in respiratory tract infections. This is important because MMP patients are predominantly elderly, with the disease mostly diagnosed in patients over 60 years, and are usually already under aggressive immunosuppression when RTX is applied (6). Our results suggest that RTX does not further add to the long-term risk of MMP patients for infections. Thus, the decision to use RTX in MMP patients is not a trade-off between safety and disease control. This good tolerability of RTX in MMP patients is in line with a recent retrospective study in patients with bullous pemphigoid (BP) who have similar demographics and are immunosuppressed to a degree similar to MMP patients. This study showed that the use of RTX in BP is associated with longer survival indicating a small risk for lethal infections due to RTX (10).

Collectively, considering that disease severity improved in 67% of MMP patients and that MMP is a debilitating, most difficult-to-treat disease, we therefore propose that there are more potential benefits in applying RTX to MMP patients than in withholding it. However, it must be noted that the retrospective design of our study limits its power to comprehensively determine the effectivity and risks of the use of rituximab in MMP patients.

## Limitations

The limitations of this study are that it is a monocentric, retrospective study, including only 18 patients who received rituximab. MMP is a heterogeneous disease; thus, studies on the effect of RTX and its side effects would probably benefit from the stratification of patients with different patterns of organ affection. This, however, was not possible in this study with its low number of patients. Furthermore, the treatment of the patients over time did not follow a specific protocol, and there is no control group of MMP patients not receiving rituximab.

## Data availability statement

The original contributions presented in the study are included in the article/**Supplementary Material**. Further inquiries can be directed to the corresponding author.

## Ethics statement

The studies involving human participants were reviewed and approved by Ethics committee of the University of Lübeck. The patients/participants provided their written informed consent to participate in this study.

## Author contributions

FB, DZ and CS conceived the study. FB retrieved and collected the data. FB, IK, DG and CS analyzed the results. FB and CS wrote the manuscript and prepared the figures. IK, DG and DZ edited the manuscript. All authors contributed to the article and approved the submitted version.

## In memoriam

This article is part of the memorial collection for Detlef Zillikens. This research project was inspired by a discussion the first author and the senior author had with Detlef Zillikens during a ward round. This article was the last manuscript Detlef Zillikens worked on while being on sick leave in March 2022 due to an unknown condition, and at this point, he still had not anticipated that he would not return to work and would pass away only 6 months later.
