# Supplementary material for: Retrospective analysis of the long-term therapeutic effectiveness and safety profile of rituximab in the treatment of mucous membrane pemphigoid in a German university center between 2008 and 2019
Source: Front Immunol. 2023 Apr 18;14:1180150. doi: 10.3389/fimmu.2023.1180150 (PMC10153668; doi:10.3389/fimmu.2023.1180150)
Supplement: Supplementary file 1 [file Table_1.docx]

**Table S1. Definitions of disease activity according to Murrell et al. [1] and own added definitions.**

| Early observation points | | | |
| --- | --- | --- | --- |
|  | Disease control | | |
|  |  | The time at which new inflammatory lesions cease to form and established lesions begin to heal. | |
| Late observation points | | | |
|  | Partial remission | | |
|  |  | Partial remission on therapy* | |
|  |  |  | The presence of transient new lesions that heal without scarring within 1 week while the patient is receiving more than minimal therapy for at least 2 months. |
|  |  | Partial remission on minimal therapy | |
|  |  |  | The presence of transient new lesions that heal without scarring within 1 week while the patient is receiving minimal therapy for at least 2 months. |
|  |  | Partial remission off therapy | |
|  |  |  | Presence of transient new lesions that heal within 1 week without treatment while the patient is off all MMP therapy for at least 2 months. |
|  | Complete remission | | |
|  |  | Complete remission on therapy* | |
|  |  |  | The absence of new or established lesions while the patient is receiving more than minimal therapy for at least 2 months. |
|  |  | Complete remission on minimal therapy | |
|  |  |  | The absence of new or established lesions while the patient is receiving minimal therapy for at least 2 months. |
|  |  | Complete remission off therapy | |
|  |  |  | Absence of new or established lesions while the patient is off all MMP therapy for at least 2 months. |
| Relapse | | | |
|  | Appearance of ≥ 3 new lesions a month (blisters, erosions) that do not heal within 1 week, or the extension of established lesions in a patient who has achieved disease control. | | |

*, own added definitions

**Reference**

[1] Murrell DF, Marinovic B, Caux F, Prost C, Ahmed R, Wozniak K, et al. Definitions and outcome measures for mucous membrane pemphigoid: recommendations of an international panel of experts. J Am Acad Dermatol. 2015;72; 168-174.

| **Patient No.** | **Possible Adverse Events**  **Table S2. Possible adverse events 12 months before and after RTX.** | | **Months after RTX** | **Follow-up (months)** | |
| --- | --- | --- | --- | --- | --- |
|  | **Before RTX** | **After RTX** |  | **Before RTX** | **After RTX** |
| 1 | Unknown | None | - | 0 | 12 |
| 2 | None | Muscular cramps | 5 | 2 | 12 |
|  |  | Osteoporosis | 10 |  |  |
|  |  | Arthrosis | 11 |  |  |
| 3 | None | Upper respiratory infection | 3 | 9 | 12 |
| 4 | Bronchitis | Upper respiratory infection | 3 | 4 | 12 |
| 5 | None | Pneumonia | 1 | 12 | 10 |
|  |  | Upper respiratory infection | 5 |  |  |
| 6 | Hepatits B infection | None | - | 12 | 12 |
|  | Upper respiratory infection |  |  |  |  |
| 7 | Urinary tract infection | Gastrointestinal infection | 10 | 6 | 12 |
|  | Orthostatic circulatory dysregulation |  |  |  |  |
|  | Upper respiratory infection |  |  |  |  |
| 8 | None | Fibromyalgia syndrome | 1 | 1 | 12 |
|  |  | Upper respiratory infection | 6 |  |  |
|  |  | Sepsis | 6 |  |  |
| 9 | Urinary tract infection | Urinary tract infection | 1 | 12 | 11 |
| 10 | Urinary tract infection | None | - | 4 | 12 |
| 11 | Genital herpes | Bronchitis | 2 | 5 | 5 |
|  | Upper respiratory infection |  |  |  |  |
|  | Gastrointestinal infection |  |  |  |  |
|  | Pulpitis |  |  |  |  |
| 12 | Upper respiratory infection | None | - | 3 | 12 |
| 13 | Urinary tract infection | Osteopenia | 2 | 12 | 12 |
|  |  | Progressive cataract formation | 4 |  |  |
| 14 | None | Myalgia | 0 | 7 | 12 |
| 15 | Unknown | Urinary tract infection | 4 | 0 | 12 |
|  |  | Upper respiratory infection | 6 |  |  |
| 16 | None | Asthma exacerbation | 0 | 12 | 12 |
|  |  | Dyspnoea | 0 |  |  |
|  |  | Reduced ejection fraction (30%) | 3 |  |  |
|  |  | Left bundle branch block | 3 |  |  |
|  |  | Macular foramen | 3 |  |  |
|  |  | Vitreous opacity | 3 |  |  |
| 17 | None | None | - | 2 | 12 |
| 18 | Unknown | None | - | 0 | 12 |

| **Pat. No.**  **Table S3. Demographics, autoantigens, and organ involvement** | **Sex** | **Age*** | **Co-morbidities** | **Autoantibodies** | | **Organs affected before RTX** |
| --- | --- | --- | --- | --- | --- | --- |
|  |  |  |  | **Target antigen** | **Isotype(s)** |  |
| **1** | M | 46 | Hypothyroidism  Coronary heart disease  Slipped disk | Laminin-332 | IgG | Oral mucosa  Nasal mucosa  Ocular mucosa |
| **2** | M | 58 | Slipped disk  Eye surgery after trauma | BP180 | IgA | Oral mucosa  Nasal mucosa  Ocular mucosa |
| **3** | M | 90 | Myocardial infarction  Arterial hypertension Hyperlipidemia | LAD-1 | IgG + IgA | Oral mucosa  Nasal mucosa  Genital mucosa |
| **4** | F | 68 | Arterial hypertension  T2D  Adipositas per magna Coxarthrosis | BP180 | IgG | Genital mucosa  Anal mucosa  Skin |
| **5** | F | 62 | Multiple sclerosis Hashimoto thyroiditis | LAD-1 | IgG + IgA | Oral mucosa  Nasal mucosa  Genital mucosa |
| **6** | M | 72 | Urothelial carcinoma  Gastric ulcer | Laminin-332 | IgG | Oral mucosa  Nasal mucosa  Laryngeal mucosa |
| **7** | F | 24 | Stomatitis | BP180 | IgG | Oral mucosa  Ocular mucosa |
| **8** | F | 39 | Arterial hypertension  T2D  Adipositas  Steatosis hepatis | BP230 | *Unknown* | Oral mucosa |
| **9** | F | 67 | T2D  Atrial fibrillation  Arterial hypertension Depression  Slipped disk | *Unknown* | *Unknown* | Ocular mucosa |
| **10** | F | 76 | None | LAD-1 | *Unknown* | Oral mucosa |
| **11** | F | 55 | Atopic dermatitis Psoriasis capitis Hypothyroidism | BP180 | *Unknown* | Oral mucosa |
| **12** | F | 39 | Arterial hypertension | LAD-1 | IgA | Oral mucosa  Nasal mucosa |
| **13** | F | 74 | None | BP180  BP230 | IgG | Oral mucosa  Nasal mucosa  Genital mucosa  Skin |
| **14** | F | 74 | None | LAD-1 | IgA | Oral mucosa  Ocular mucosa |
| **15** | F | 77 | Psoriasis vulgaris  Hashimoto thyroiditis Arthrosis  COPD | Laminin-332 | *Unknown* | Ocular mucosa  Anal mucosa |
| **16** | M | 71 | None | BP180 | IgG | Oral mucosa  Nasal mucosa |
| **17** | F | 55 | Cervical carcinoma | Laminin-332 BP180 | IgG | Oral mucosa |
| **18** | F | 36 | None | *Unknown* | IgG | Oral mucosa |

*, at first administration of RTX; F, female; M, male; T2D, type 2 diabetes mellitus

| **Pat. No.** | **Systemic treatments** | | |
| --- | --- | --- | --- |
|  | **At initiation of RTX** | **Up to 6 months**  **after RTX*** | **Month 7 – 12 after RTX*** |
| **1** | DX pulses *(3 x 100 mg each)*  CP pulses *(750 mg each)*  Protein A-IA | DX pulses *(3 x 100 mg each)*  CP pulses *(750 mg each)*  MMF *(2000 mg/d)*  Protein A-IA | DX pulses *(3 x 100 mg each)*  CP pulses *(750 mg each)*  MMF *(2000 mg/d)* |
| **2** | DX pulses *(1 x 100 mg each)*  MMF *(2000 mg/d)* | DX pulses *(1 x 100 mg each)*  Prednisone *(15 mg/d)*  MMF *(1000 mg/d)*  IVIg | Prednisone *(7.5 mg/d)*  CP pulses *(750 mg each)*  MMF *(720 mg/d)*  IVIg |
| **3** | DX pulses *(3 x 50 mg each)*  MMF *(2000 mg/d)*  IVIg | DX pulses *(3 x 50 mg each)*  MMF *(1000 mg/d)*  IVIg | DX pulses *(3 x 50 mg each)*  MMF *(1000 mg/d)*  IVIg |
| **4** | DX pulses (3 x 100 mg each)  MMF *(2000 mg/d)*  Dapsone *(50 mg/d)* | DX pulses *(3 x 100 mg each)*  MMF *(2000 mg/d)*  Dapsone *(50 mg/d)* | DX pulses *(3 x 100 mg each)*  MMF *(2000 mg/d)*  Dapsone *(75 mg/d)* |
| **5** | DX pulses *(3 x 100 mg each)*  MMF *(2000 mg/d)* | DX pulses *(3 x 100 mg each)*  MMF *(2000 mg/d)* | MMF *(2000 mg/d)*  IVIg |
| **6** | DX pulses *(3 x 100 mg each)*  MMF *(1440 mg/d)* | DX pulses *(3 x 100 mg each)*  MMF *(1440 mg/d)*  IVIg | DX pulses *(3 x 100 mg each)*  MMF *(720 mg/d)*  IVIg |
| **7** | DX pulses *(3 x 100 mg each)*  MMF *(1440 mg/d)* | DX pulses *(3 x 100 mg each)*  MMF *(1440 mg/d)* | DX pulses *(3 x 100 mg each)*  MMF *(1440 mg/d)* |
| **8** | DX pulses *(3 x 100 mg each)*  AZT *(150 mg/d)* | DX pulses *(3 x 100 mg each)*  AZT *(100 mg/d)*  IVIg | MMF *(1000 mg/d)*  IVIg |
| **9** | DX pulses *(3 x 100 mg each)*  MMF *(2000 mg/d)*  IVIg | DX pulses *(2 x 100 mg each)*  MMF *(2000 mg/d)*  IVIg | DX pulses *(3 x 100 mg each)*  MMF *(2000 mg/d)*  IVIg |
| **10** | DX pulses *(3 x 50 mg each)*  Dapsone *(100 mg/d)* | DX pulses *(3 x 50 mg each)*  Dapsone *(100 mg/d)* | *No data available* |
| **11** | DX pulses *(3 x 50 mg each)*  MMF *(1440 mg/d)* | DX pulses *(3 x 50 mg each)*  MMF *(1440 mg/d)* | *No data available* |
| **12** | DX pulses *(3 x 100 mg each)*  MMF *(2000 mg/d)*  Dapsone *(150 mg/d)* | DX pulses *(3 x 100 mg each)*  MMF *(2000 mg/d)*  Dapsone *(150 mg/d)*  IVIg | DX pulses *(3 x 100 mg each)*  MMF *(2000 mg/d)*  Dapsone *(150 mg/d)*  IVIg |
| **13** | DX pulses *(3 x 100 mg each)*  MMF *(2000 mg/d)* | DX pulses *(3 x 100 mg each)*  MMF *(2000 mg/d)* | DX pulses *(3 x 100 mg each)*  MMF *(2000 mg/d)*  IVIg |
| **14** | DX pulses *(3 x 100 mg each)*  CP pulses *(500 mg each)*  MMF *(1000 mg/d)* | DX pulses *(3 x 50 mg each)*  MMF *(1000 mg/d)*  Dapsone *(75 mg/d)*  IVIg | MMF *(1000 mg/d)*  Dapsone *(75 mg/d)*  IVIg |
| **15** | DX pulses *(3 x 100 mg each)* | DX pulses *(3 x 100 mg each)*  Dapsone *(100 mg/d)*  IVIg | DX pulses *(3 x 50 mg each)*  Dapsone *(100 mg/d)*  IVIg |
| **16** | DX pulses *(3 x 100 mg each)*  Dapsone *(100 mg/d)* | DX pulses *(3 x 50 mg each)*  Prednisone *(5 mg/d)*  Dapsone *(100 mg/d)* | Dapsone *(75 mg/d)* |
| **17** | DX pulses *(3 x 75 mg each)* | DX pulses *(3 x 75 mg each)*  Prednisone *(30 mg/d)* | DX pulses *(3 x 75 mg each)* |
| **18** | DX pulses *(3 x 50 mg each)*  MMF *(1440 mg/d)*  Protein A-IA | DX pulses *(3 x 100 mg each)*  MMF *(1440 mg/d)*  Protein A-IA | DX pulses *(3 x 100 mg each)*  MMF *(1440 mg/d)* |

**Table S4. Systemic treatments for MMP in the individual patients before and after RTX**

*, the last dose administered during this period is indicated; DX, dexamethasone; MMF, mycophenolate (mofetil); CP, cyclophosphamide; IA, immunoadsorption; IVIg, intravenous immunoglobulins; AZT, azathioprine

**Table S5. Intervals between dexamethasone pulses before and after RTX**

| **Pat. No.** | **Interval between dexamethasone pulses (weeks)** | |
| --- | --- | --- |
|  | **Before RTX** | **6 months after RTX** |
| **1** | 3 | 6 |
| **2** | 4 | discontinued |
| **3** | 3 | 6 |
| **4** | 3 | 5 |
| **5** | 7 | discontinued |
| **6** | 4 | 7 |
| **7** | 5 | 7 |
| **8** | 6 | discontinued |
| **9** | 4 | 5 |
| **10** | 5 | *No data available* |
| **11** | 6 | *No data available* |
| **12** | 6 | 10 |
| **13** | 4 | 5 |
| **14** | 4 | discontinued |
| **15** | 4 | 5 |
| **16** | 5 | discontinued |
| **17** | 4 | 7 |
| **18** | 3 | 4 |
